# Protein Induced by Vitamin K Absence II (PIVKA-II) as a potential serological biomarker in pancreatic cancer: a pilot study

**DOI:** 10.11613/BM.2019.020707

**Published:** 2019-06-15

**Authors:** Sara Tartaglione, Irene Pecorella, Serena Rita Zarrillo, Teresa Granato, Valentina Viggiani, Lucia Manganaro, Cinzia Marchese, Antonio Angeloni, Emanuela Anastasi

**Affiliations:** 1 Department of Molecular Medicine, Policlinico Umberto I, University of Rome “Sapienza”, Rome, Italy; 2 Department of Radiological Sciences, Oncology and Anatomical Pathology, Policlinico Umberto I, University of Rome “Sapienza”, Rome, Italy; 3 CNR-IBPM, National Research Council, Rome, Italy; 4 Department of Experimental Medicine, Policlinico Umberto I, University of Rome “Sapienza”, Rome, Italy

**Keywords:** pancreatic cancer, PIVKA-II, CA 19-9, CA 242, CEA

## Abstract

**Introduction:**

Protein induced by vitamin K absence II (PIVKA-II) is an abnormal prothrombin increased in gastrointestinal malignancy. We aimed to evaluate PIVKA-II in comparison to established pancreatic cancer (PC) biomarkers (CA 19-9, carcinoembryonic antigen (CEA) and CA 242) measured in PC patients and in patients with benign pancreatic diseases.

**Materials and methods:**

We studied 26 PC patients (Group 1) and 20 patients with benign pancreatic diseases (Group 2). PIVKA-II and CEA were measured by chemiluminescent enzyme immunoassay method (CLEIA) on LUMIPULSE G1200 (Fujirebio-Europe, Gent, Belgium), CA 19-9 and CA 242 were measured by ELSA (CisBio Bioassays, Codolet, France) and EIA (Fujirebio Diagnostics AB, Göteborg, Sweden), respectively. Receiver operating characteristic (ROC) analysis was performed to assess biomarkers’ diagnostic characteristics in both groups.

**Results:**

Median and interquartile range (IQR) in Group 1 and Group 2 were: 1749.0 (320.2 – 3921.0) *vs*. 31.0 (23.0 – 43.0) mAU/mL (P < 0.001) for PIVKA-II, 260.0 (158.7 – 272.0) *vs*. 45.2 (9.0 – 58.0) U/mL (P = 0.034) for CA 19-9, 104.0 (30.2 – 150.0) *vs*. 7.2 (4.8 – 26.0) U/mL (P < 0.050) for CA 242, 9.4 (5.3 – 37.5) *vs*. 4.5 (1.8 – 7.0) ng/mL (P = 0.021) for CEA. Areas under the ROC curve of PIVKA-II, CA 19-9, CA 242, CEA were 0.86 (95% CI: 0.71 – 1.00), 0.58 (95% CI: 0.38 – 0.78), 0.73 (95% CI: 0.54 – 0.92), 0.64 (95% CI: 0.44 – 0.85), respectively.

**Conclusions:**

PIVKA-II is significantly higher in PC than in benign pancreatic diseases. PIVKA-II shows a rather good diagnostic performance compared to CA 19-9, CEA and CA242, thus its determination could help PC management.

## Introduction

Pancreatic cancer (PC) is the fourth cause of cancer–related death worldwide, despite being the thirteenth most frequent neoplasm (*1*). When PC is identified in stage I, patients have 25% of 5-year survival rate. Unfortunately, 80% of patients are diagnosed at an advanced or metastatic stage of disease, when the 5-year survival is 2% (*2*). In spite of recent progresses in the clinical management of PC, its overall survival rate has not raised during the last two decades (*3*). Taken together, these observations highlight the need to identify early detection tumour biomarkers for PC that would greatly affect patient management and prognosis. At present, many serum tumour biomarkers (such as CA 19-9, CA 242, carcinoembryonic antigen (CEA), *etc.*) have been proposed for PC detection, even if the benefits of these biomarkers are unclear, since although sensitivity is increased, specificity is often not adequate (*4*). CA 19-9, an epitope of sialylated Lewis blood group antigen on Mucin 1 (MUC-1) is commonly expressed by PC cell: the majority of recommendations on early diagnosis of PC established to use serum CA 19-9 determination as a complementary test (*5*). Currently this serum biomarker is used widespread to assess disease progression in PC, but is not recommended for general screening since its serum concentration is also high in non-neoplastic pancreatic conditions (*i.e*. chronic pancreatitis) and it can produce false negatives too (*6*).

CA 242, a carbohydrate antigen, is considered a useful biomarker in PC since high serum CA 242 concentrations have been significantly associated with the diagnosis of PC (*7*). Even if there is not an elevation of CA 242 in serum in conditions such as acute pancreatitis or cholestasis (unlike CA19-9), positive CA 242 has been demonstrated not only in PC but also in colon cancer (*8*).

Carcinoembryonic antigen is one of the most widely used tumour biomarkers, it is a glycoprotein often measured for diagnosing neoplasms of digestive system (*5*). Several studies have reported that it could have an important role in predicting survival of PC patients, but the relationship between CEA and PC is undetermined yet because CEA elevation has been reported in other different gastrointestinal adenocarcinomas like gastric and colon cancer (*9*). Actually, there are no other tumour-specific markers strictly recommended for diagnosing PC but there is a persistent research for new biomarkers to facilitate earlier identification of this malignancy. Recently, a rising attention has been focusing on the relationship between vitamin K and malignancy. Regarding PC, multiple studies have confirmed that apoptosis has a key role in vitamin K-induced pancreatic cell death and the contribution of vitamin K against PC cell oncogenesis has been recently evaluated, with all studies having the common topic of apoptosis (*10*, *11*). In the light of these recent articles, we decided to investigate the role of prothrombin induced by vitamin K absence II (PIVKA-II). Prothrombin induced by vitamin K absence II is also known as des-γ-carboxy prothrombin, and is an abnormal form of prothrombin released by the liver in case of vitamin K insufficiency or as consequence of an acquired defect in the post-translational carboxylation of the prothrombin precursor in cancer cells (*12*). It has been well demonstrated in literature that PIVKA-II serum concentrations are increased in hepatocellular carcinoma (HCC). This molecule is presently an important biomarker in the diagnosis and screening for this type of neoplasm (*13*). Furthermore, rise in PIVKA-II above normal limits has been recently reported not only in HCC but also in other gastrointestinal malignancies, including PC (*14*-*16*). The present study aimed to evaluate the potential role of PIVKA-II in PC: we compared its serum concentration to other already established tumour markers (CA19-9, CEA and CA 242) measured in patients affected by PC and in patients with benign pancreatic diseases.

## Materials and methods

### Study design and subjects

This research was designed as retrospective observational study. From January 2016 to December 2017 we collected and analysed 26 serum samples of patients (12 males, 14 females, age range: 50 – 92) with PC (Group 1), and 20 serum samples of patients (12 males, 8 females, age range: 39 – 85) with benign pancreatic diseases (Group 2). All patients were referred to the Oncologic Unit A, of the Policlinico Umberto I, Rome, Italy. Group 1 patients met the following eligible criteria: adult age (≥ 18 years), the first occurrence of neoplastic pathology, no prior treatment with neoadjuvant therapy, absence of diabetes, no serious physical disabilities. The criteria for inclusion to Group 2 were: adult age (≥ 18 years), absence of any present malignant tumour, no prior occurrence of neoplastic pathology, no diabetes, no serious physical disabilities, presence of a pancreatic benign disease (pancreatitis, pancreatic benign cystic lesions, benign pancreatic neoplasms). The study protocol was approved by the Institutional Review Board and all subjects participating in the study, patients and volunteers signed a written informed consent. At enrolment, medical history was collected for each patient, and peripheral blood samples were drawn and immediately sent to the laboratory of Tumour Markers of the Policlinico Umberto I, Rome. When diagnosis was made for every participant in the study, each serum sample was analysed for PIVKA-II, CA19-9, CEA and CA 242. Group 1 and Group 2 patients’ diseases were confirmed by histopathological examination conducted in the Histopathological Unit of the Policlinico Umberto I, Rome, Italy. All Group 1 patients were subjected to postoperative histopathological diagnosis, which confirmed the presence of PC (4 in stage II B, 2 in stage II A, 12 in stage III and 8 patients in stage IV). The research was performed in compliance with the current revision of Helsinki declaration.

### Blood sampling

Blood collection was performed following a standard protocol. Peripheral blood samples were obtained by venous puncture, collected in a red top Vacutainer (Becton, Dickinson and Company, Plymouth, UK) clotted 60 – 90 minutes and centrifuged for 10 minutes at 1300xg. The serum fractions were aliquoted in 1.5 mL Eppendorf tubes (Eppendorf srl, Milano, Italy) and stored at – 80 °C until analysis.

### Methods

#### PIVKA-II and CEA

Prothrombin induced by vitamin K absence II and CEA serum concentrations were determined on a Lumipulse G1200 (Fujirebio-Europe, Gent, Belgium), using the LUMIPULSE G PIVKA-II kit and the LUMIPULSE G CEA kit (Fujirebio, Tokyo, Japan) respectively (*17*). Lumipulse G1200 is a fully automated assay system for the quantitative measurement in serum specimen based on chemiluminescent enzyme immunoassay (CLEIA) technology by a two-step sandwich in immunoreaction cartridges. For CEA the detection range was between 0.5 – 200 ng/mL, with intra-assay coefficient of variation (CV) of < 2.4%, inter-assay CV of < 10% and a cut-off of 6 ng/mL (CV < 10%) based on the 95% confidence interval, according to manufacturer specifications. For PIVKA-II the detection range was between 5 - 75,000 mAU/mL, with intra-assay CV of < 2.4% and inter-assay CV of < 10% and as a clinical cut-off we considered 48 mAU/mL. We defined our internal cut-off within our laboratory because there is a discordant literature between Japanese and American studies on the cut-off to consider (*17*).

#### CA 19-9

CA 19-9 serum concentration was quantified by a manual radioimmunoassay (RIA) method (ELSA-CA19-9, CisBio Bioassays, Codolet, France). The diagnostic kit RIA ELSA-CA 19-9 CisBio is a solid-phase two-step “sandwich” immunometric assay. The detection range was between 1.5 and 240 U/mL, with intra-assay CV of < 3.8%, inter-assay CV of < 4.0% and a cut-off value of 37 U/mL (CV < 10%) based on the 95% confidence interval according to manufacturer’s specifications.

#### CA 242

CA 242 concentration in serum specimens was measured by a manual enzyme immunoassay (EIA) technique using the CanAg CA242 EIA kit (Fujirebio Diagnostics AB, Göteborg, Sweden). The CanAg CA242 EIA is a solid phase, non-competitive immunoassay. The detection range was between 1 and 150 U/mL, with intra-assay CV of < 3.8% and inter-assay CV of < 4.0% According to manufacturer was considered a cut-off of normality of 16 U/mL (CV < 10%) based on the 95% confidence interval.

All assays were performed in duplicate and according to the manufacturers’ instructions.

### Statistical analysis

Since sample size in our study was < 30, a non-parametric Mann Whitney U test was performed to determine the differences in accuracy of PIVKA-II, CA 19-9, CEA and CA 242 for PC versus benign pancreatic diseases (*18*). The results are expressed as median and interquartile ranges (IQR). To evaluate the discrimination ability of the tested biomarkers, area under the receiver operating characteristic curve (ROC AUC) were calculated. For each AUC we estimated the 95% confidence interval (95% CI). The values P < 0.05 were considered statistically significant. Statistical analysis was performed using StatsDirect 3.0.187 statistical software (StatsDirect software, Cheshire, England)

## Results

**Figure 1:**
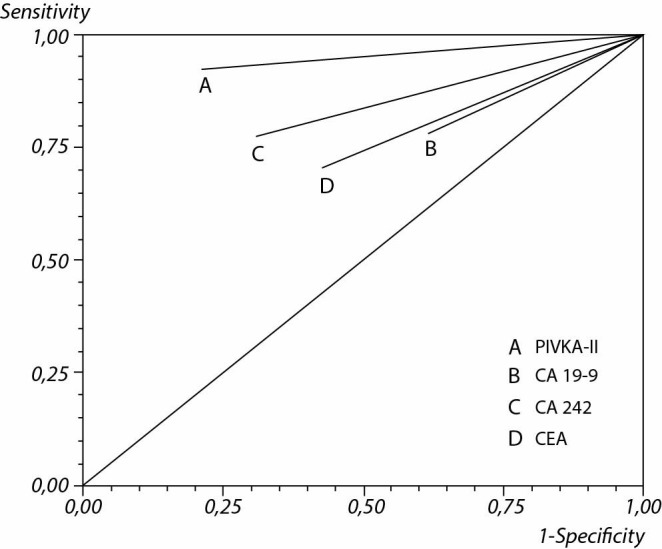
Receiver operating characteristic curves analysis of PIVKA-II, CA19-9, CA242 and CEA.

Prothrombin induced by vitamin K absence II, CA 19-9, CA 242 and CEA serum concentrations were evaluated in Group 1 and Group 2. Results expressed as median and IQR ranges are presented in Table 1. Clinical cut-off for PIVKA II was 48 mAU/mL, according to our internal measurements. All examined tumour markers’ concentrations were significantly higher in PC than benign pancreatic diseases. Tested differences with P values are presented in Table 1. In Group 1, 24/26 patients had high PIVKA-II in comparison with the cut-off established within our laboratory while both CA 19-9 and CA 242 concentrations were above the cut-off in 20/26 of the cases, and CEA was positive only in 16/26. Conversely, in Group 2 we observed high PIVKA-II concentrations in comparison with our cut-off in just 4/20 of patients, while CA 19-9, CA 242 and CEA were positive in 12/20, 6/20 and 8/20 of patients respectively. Receiver operating characteristic curves analysis were used to assess the performance of the PIVKA-II, CA 19-9, CA 242 and CEA in discriminating PC from benign pancreatic diseases (Table 2). PIVKA-II had a large AUC and showed an optimal sensitivity (92%) and a quite good specificity (80%) while CA 242 had an acceptable sensitivity (77%) but rather low specificity (70%) (Figure 1).

**Table 1:** Tumour markers in pancreatic cancer and benign pancreatic disease patient group

**Tumour marker, unit**	**Pancreatic cancer** **(N = 26)**	**Bening pancreatic lesions** **(N = 20)**	**P**
**PIVKA II, mAU/mL**	1749.0 (320.2 – 3921.0)	31.0 (23.0 – 43.0)	< 0.001
**CA 19-9, U/mL**	260.0 (158.7 – 272.0)	35.2 (9.0 – 58.0)	0.034
**CA 242, U/mL**	104.0 (30.2 – 150.0)	7.2 (4.8 – 26.0)	0,048
**CEA, ng/mL**	9.4 (5.3 – 37.5)	4.5 (1.8 – 7.0)	0.021
Data are presented as median and interquartile range. Continuous variables were compared using Mann Whitney test. P < 0.05 was considered statistically significant.			

**Table 2:** Receiver operating characteristic curves analyses for the investigated tumour markers

**Tumour marker**	**Sensitivity**	**Specificty**	**AUC**
**PIVKA II**	0.92 (0.64 – 0.99)	0.80 (0.44 – 0.97)	0.86 (0.71 – 1.00)
**CA 19-9**	0.77 (0.46 – 0.95)	0.40 (0.12 – 0.74)	0.58 (0.38 – 0.78)
**CA 242**	0.77 (0.46 – 0.95)	0.70 (0.38 – 0.93)	0.73 (0.54 – 0.92)
**CEA**	0.69 (0.38 – 0.90)	0.60 (0.26 – 0.88)	0.64 (0.44 – 0.85)
Sensitivity, specificity and area under the curve (AUC) are presented in percentage with corresponding 95% confidence intervals.			

## Discussion

In the present study, we reported for the first time that PIVKA-II is positive in a cohort of PC Italian patients and its median serum concentrations are significantly higher in PC than in benign pancreatic diseases. According to ROC curve analysis, PIVKA II had a large AUC, which combined remarkable sensitivity and specificity in the differentiation of PC and benign pancreatic diseases. Additionally, we found that the median serum concentrations of CA 19-9, CA 242 and CEA were all higher in PC than in benign pancreatic diseases, but according to their AUCs only CA 242 showed a good diagnostic performance.

In the last years, many studies have provided evidences on the role of PIVKA-II as a biomarker for gastrointestinal malignancies: particularly, it has been well demonstrated in literature that PIVKA-II is biomarker of crucial importance in HCC since its serum values are linked to the cancer volume, potential of microvascular metastasization and can predict tumour recurrence (*19*). Pancreas originates from the primitive foregut of the embryo like the liver and is therefore largely believed that these two organs have a quiescent capability to transdifferentiate into each other’s tissue. Consequently, PIVKA-II, which is characteristic of HCC, can credibly be expressed in PC, even if the mechanism of PIVKA-II pancreatic production is still undetermined. In agreement with data reported in literature we hypothesized that the common foregut derivation of liver and pancreas, their embryologic proximity and their demonstrated capability of mutual trans-differentiation could explain a possible PIVKA-II production by PC cells (*20*, *21*). Additionally, it is known that PIVKA-II is produced by the liver not only in presence of neoplastic cells, but also in absence of vitamin K (*12*). Various *in vitro* and *in vivo* studies have demonstrated anti-carcinogenic effects played by vitamin K both directly (due to its ability to suppress cancer growth and cause apoptosis in neoplastic cells) and indirectly through post-translational activation of proteins including PIVKA-II, which have thus been proposed as tumour markers for diverse types of malignancies (*10*). According to these findings, the hypothetical mechanism we speculate for PIVKA-II high concentrations in PC is that they could reflect a possible association between vitamin K status and PC.

CA 19-9 is currently the most important biomarker for PC but its serum concentrations are more useful for monitoring responses to therapy rather than in early diagnosis (*22*). In literature, positive CA 19-9 was reported only in 50% of PC patients (*23*). CA 19-9 accuracy varies with disease stage and as a diagnostic biomarker it lacks sufficient sensitivity and specificity: the antigen is not expressed in 5% to 10% of patients with fucosyl transferase deficiency and ineffectiveness to synthesize antigens of the Lewis blood group but it is conversely found on epithelia of normal pancreas, biliary duct, stomach and colon (*24*, *25*). Accordingly, in our population CA 19-9 revealed a bad diagnostic performance since its AUC was small and not statistically significant.

CA 242 on contrary had a good diagnostic performance in our population, according to its AUC. CA 242 is a serological marker increased in PC patients, its serum values have been found to have a correlation with cancer size and differentiation, lymph node and liver metastasis status and clinical staging (*26*). Recently a meta-analysis has shown that the combined tests of CA 19-9 plus CA 242 could have a more effective diagnostic value than individual (*27*). As reported by Gui in 2014, in our study we found that CA 242 specificity is higher than that of CA 19-9 (*28*). Moreover, as we shown in our population, CA 242 specificity is still suboptimal (*29*).

Carcinoembryonic antigen is the second most commonly used biomarker for PC detection despite its pathological values are found only in 30-60% of PC patients, data confirmed also in our population (*30*). Carcinoembryonic antigen when combined with CA 19-9 can improve the accuracy in distinguishing neoplastic from normal patients but it has still a limited sensitivity. Increased CEA serum concentration is in fact generally found in adenocarcinomas, including stomach cancer and colon cancer (*29*). However, in line with our findings, there is a large body of evidence that define sensitivity and specificity of CEA in PC as not optimal also because CEA can frequently be positive in diverse non neoplastic conditions such as non-specific colitis and nicotine addiction (*27*). When referring to CEA in our study, it revealed a bad diagnostic performance since its AUC was small and not statistically significant.

Because of the insufficient individual sensitivity or specificity of already established PC biomarkers, our study is part of the constant ongoing effort to identify additional serological biomarkers for the timely detection of PC. Recent studies have been focusing on the discovery of new biomarkers that would facilitate PC identification but to our knowledge, this pilot study is the first to explore the role of PIVKA-II as a biomarker for PC. However, our results are in line with a reported case of a PIVKA-II producing PC (*16*). Our data also suggests that the evaluation of serum PIVKA-II appears to be useful not only for PC detection but also for differential diagnosis of pancreatic lesions. In our study, this biomarker in fact resulted less prone to elevation above the cut-off in case of benign pancreatic diseases than currently used biomarkers for PC like CA19-9, CEA and CA 242. In conclusion, since PIVKA-II is significantly higher in PC than in benign pancreatic diseases and shows a quite good diagnostic performance compared to CA19-9, CEA and CA 242, its determination could be considered in the clinical management of PC. Despite the fact that our results are very promising, we are aware of the limitations of this study. This was a single centre study, the sample size was small, the tumour staging was not the same for all Group 1 patients, pancreatic benign disease were heterogeneous. For these reasons, we consider it as a preliminary study: further large-scale and multi center studies are needed to confirm the usefulness of PIVKA-II in PC detection alone or in combination with already established biomarkers.
